# Epidemiology, Clinicopathological Features, Etiological Spectrum and Outcomes of Subcutaneous Mycoses: A 14-Year Retrospective Study

**DOI:** 10.3390/jof12090692

**Published:** 2026-09-16

**Authors:** Qiannan Jia, Aiping Fan, Xiuli Xie, Jun Li, Li Zhang, Qiwen Yang

**Affiliations:** 1 Department of Dermatology, State Key Laboratory of Complex Severe and Rare Diseases, Peking Union Medical College Hospital, Chinese Academy of Medical Sciences and Peking Union Medical College, Beijing 100730, China; 2 Department of Laboratory Medicine, Peking Union Medical College Hospital, Chinese Academy of Medical Sciences and Peking Union Medical College, Beijing 100730, China

**Keywords:** subcutaneous mycoses, sporotrichosis, fungal culture, histopathology, metagenomic next-generation sequencing, prognosis

## Abstract

Background: Subcutaneous mycoses are a group of chronic deep fungal infections involving the skin and subcutaneous tissue. In this study, we characterized their epidemiology, diagnostic findings, treatment, and outcomes in a referral cohort from Beijing, China. Methods: We retrospectively reviewed 86 patients diagnosed with subcutaneous mycoses at a referral center in Beijing which serves a notable population from northeastern agricultural regions, from 2012 to 2025. Data on clinical presentations, microbiological and histopathological findings, diagnostic methods, treatment regimens, and outcomes were collected and analyzed. Results: A total of 86 patients were included, comprising 35 males (40.7%) and 51 females (59.3%). The median disease duration was 9.0 months. A history of trauma was recorded in 19 patients (22.1%). *Sporothrix* spp. were the predominant pathogens, accounting for 47 cases (54.7%), followed by filamentous fungi in 24 cases (27.9%), and yeasts and yeast-like fungi in 15 cases (17.4%). Underlying conditions affecting host defense were present in 22 patients (25.6%) and were most frequent in the yeast/yeast-like group (73.3%; *p* < 0.001). Histopathology was performed in 70 patients; fungal structures were seen in only 11.4%. Among 85 patients included in the outcome analysis, 72 (84.7%) achieved complete response. Complete-response rates differed among etiological groups (*p* = 0.004), and filamentous fungal infection was independently associated with non-complete response (OR, 6.46; 95% CI, 1.45–39.25; *p* = 0.014). Patients with underlying conditions affecting host defense had a lower complete-response rate than those without such conditions (66.7% vs. 90.6%; *p* = 0.014). Conclusions: The etiological spectrum of subcutaneous mycoses in this referral cohort was strongly influenced by epidemiological and host factors, with *Sporothrix* spp. predominating. Fungal culture remains central to etiological confirmation, while histopathology and mNGS provide complementary information. Pathogen category and host-defense status may help guide treatment stratification and follow-up.

## 1. Introduction

Subcutaneous mycoses are a heterogeneous group of infections caused by environmental fungi that are directly inoculated through traumatic implantation [1]. These infections primarily involve the dermis and subcutaneous tissues, but they may extend to adjacent structures such as the lymphatics, bone, and joints [2]. Similar to other fungal infections, immunosuppressed patients face elevated risks of developing these mycoses, with potential for widespread dissemination [3]. Characterized by subacute or chronic progression, subcutaneous mycoses are associated with considerable morbidity [4,5]. The various types of subcutaneous mycoses include sporotrichosis, mycetoma, chromoblastomycosis, phaeohyphomycosis, lobomycosis, rhinosporidiosis, and subcutaneous zygomycosis.

The global epidemiology of subcutaneous mycoses exhibits marked geographical variations, influenced by climate, environmental factors, and occupational exposures. These infections are most prevalent in tropical and subtropical regions of the world and usually affect populations in developing countries and rural areas [2,6]. Regarding occupational risk groups, these mycoses usually impact individuals involved in outdoor activities such as agriculture, hunting, mining, and lumbering [7]. In recent decades, the global burden of subcutaneous mycoses has escalated due to the growing use of antimicrobial agents, chemotherapy, immunomodulators, and transplantation therapies [8].

The diagnosis of subcutaneous mycoses relies on clinical presentation, histopathological examination, and culture of the etiologic agents [9]. Clinical manifestations of subcutaneous mycoses depend on host-related factors, fungal species, and transmission routes [9]. Presentations range from papules, plaques, and nodules to necrotic, suppurative, or ulcerative lesions, which may be solitary or multiple [6]. Such nonspecific features often complicate diagnosis, leading to misdiagnosis and delayed treatment. The isolation and identification of the etiological agents remain the key diagnostic methods of subcutaneous mycoses. However, fungal growth is often time-consuming and may need several weeks for visible colony formation [10]. Furthermore, the sensitivity of fungal culture is variable and may be limited by specimen quality, fungal load, and prior antifungal exposure, and it can also lead to unreliable results due to contamination [11,12,13,14]. In recent years, molecular techniques have increasingly complemented conventional methods, allowing for early and accurate identification of the pathogens [15]. Histopathological examinations are often suggestive but not always specific [16]. They offer a relatively rapid and reliable means of diagnosis, providing insight into tissue response and fungal localization; however, there may be discrepancies between histopathology and culture results [17], which can complicate diagnosis. Treatment typically involves antifungal therapy and/or surgical excision. Nevertheless, management of serious cases remains challenging, with reports of relapse, disease progression, and poor tolerability of antifungal drugs [3].

This study aims to review a 14-year case series of subcutaneous mycoses from a tertiary referral center in China. We analyze the epidemiological features, clinical manifestations, histopathological characteristics, microbiological findings, therapeutic strategies, and clinical outcomes, and discuss the clinical implications of our results. The findings are intended to enhance the understanding of subcutaneous mycoses and improve the diagnosis and management of these infections.

## 2. Materials and Methods

### 2.1. Patient Selection

A retrospective analysis was conducted on patients diagnosed with subcutaneous mycoses in the department of dermatology of a large referral hospital in Beijing which serves a notable population from northeastern agricultural regions, from 1 January 2012, to 31 December 2025. Ethical approval was obtained from the Institutional Ethics Committee of Peking Union Medical College Hospital (I-25PJ3339). Written informed consent was obtained from all patients or their legal guardians.

**Figure 1:**
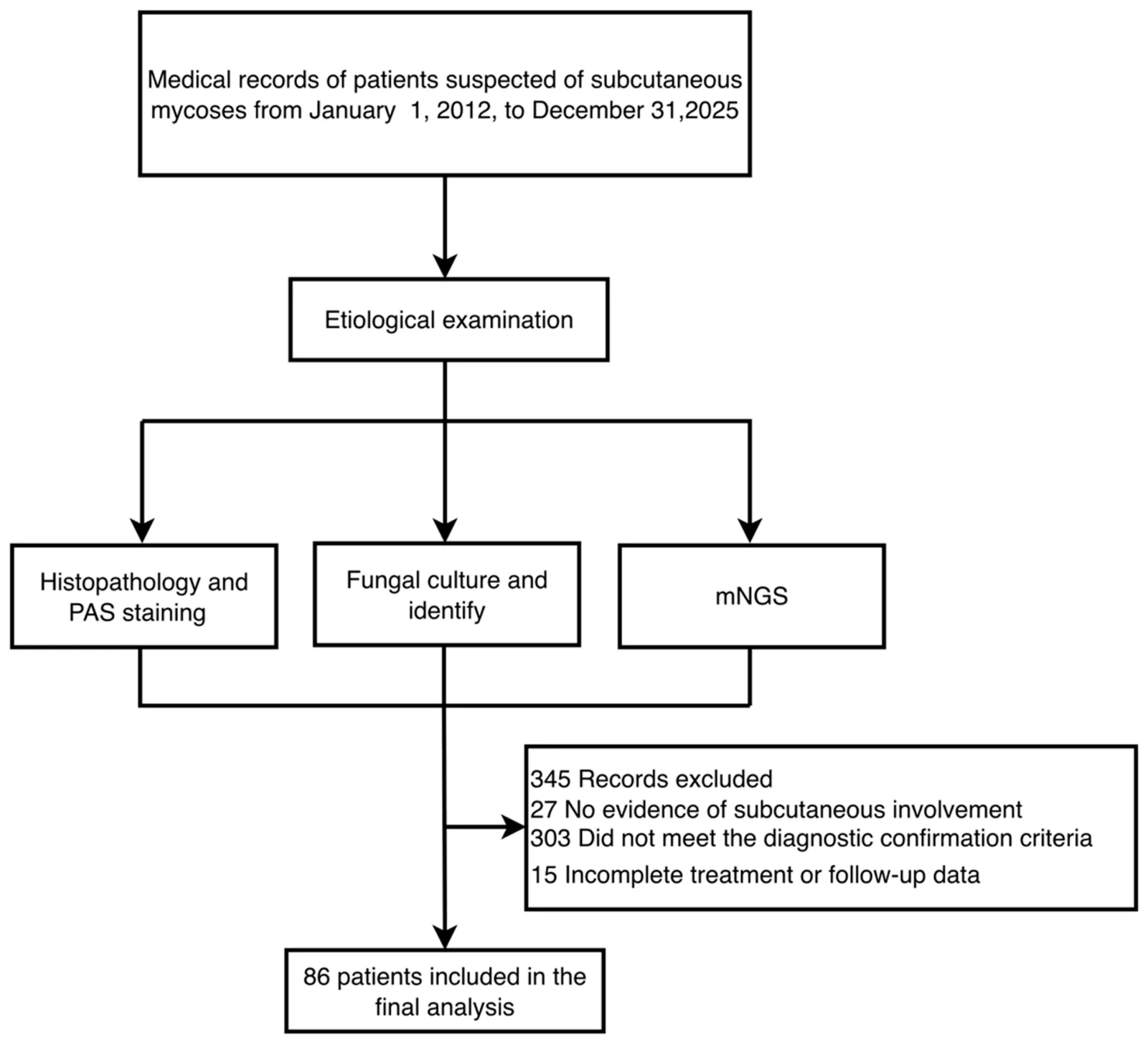
Flowchart of patient selection and study inclusion. Medical records of patients with suspected subcutaneous mycoses between 1 January 2012, and 31 December 2025, were screened. Etiological evaluation included histopathological examination with periodic acid-Schiff staining, fungal culture and identification, and metagenomic next-generation sequencing where indicated. Of the 431 records screened, 345 were excluded: 27 showed no evidence of subcutaneous involvement, 303 did not meet the diagnostic confirmation criteria, and 15 had incomplete treatment or follow-up data. Ultimately, 86 patients were included in the study. PAS, periodic acid-Schiff; mNGS, metagenomic next-generation sequencing.

Patients were included in the study only if they met all the criteria: (1) clinical suspicion of subcutaneous fungal infection; (2) at least one mycological or pathological finding supportive of fungal infection, including positive direct microscopy, positive fungal culture, histopathological evidence of fungal infection, or positive molecular identification; or (3) complete medical records including demographic information, clinical data, diagnostic procedures, treatment, and follow-up data. Patients were excluded if they had: (1) superficial fungal infections confined to the stratum corneum, hair, or nails without evidence of subcutaneous involvement; (2) insufficient clinical or laboratory data for diagnostic confirmation; or (3) incomplete medical records (Figure 1).

### 2.2. Case Data

For each patient, the following data were collected from medical records and telephone follow-up interviews: age, sex, occupation, trauma history, medical history, disease duration, lesion location, lesion morphology, direct microscopic examination, fungal culture, molecular testing, histopathological findings, treatment, and outcomes. Clinical outcomes were categorized as complete response, partial response, stable disease, relapse, or death according to documentation in the medical record and telephone follow-up interviews.

### 2.3. Mycological Examination

Clinical specimens were obtained according to the lesion type. For open lesions (e.g., ulcers, erosions), secretions or exudates were obtained through scraping. For closed lesions without exudation (e.g., nodules, plaques, or masses), skin biopsy specimens were obtained for subsequent examinations.

Samples treated with 10% KOH or fungal fluorescent staining were directly examined using a microscope. The presence of hyphae, pseudohyphae, spores, or other fungal elements was considered positive.

Specimens were inoculated on Sabouraud dextrose agar (SDA) medium supplemented with chloramphenicol and cultured at 25 °C for 2 weeks to observe colonies and microscopic morphology. Cultured isolates were initially identified by colony morphology and microscopic features, which generally supported genus-level identification. Species-level identification was assigned only when internal transcribed spacer (ITS) sequencing of the cultured isolate was available or clinically indicated, which was conducted using an ABI3730XL Genetic Analyser (Applied Biosystems, Carlsbad, CA, USA). The resulting sequences were compared against the NCBI database via the BLAST tool (http://blast.ncbi.nlm.nih.gov/Blast, accessed on various dates between 1 January 2012 and 31 December 2025).

Metagenomic next-generation sequencing (mNGS) was selectively performed in patients with negative culture, atypical manifestations, or suspected mixed infection. The results were interpreted in combination with clinical presentation, histopathology, fungal culture, and treatment response. Bacterial findings alone were not considered sufficient for inclusion, and low-read or discordant organisms without supporting evidence were considered as possible contamination or colonization.

### 2.4. Histopathological Analysis

Histopathological examination was performed on formalin-fixed, paraffin-embedded skin specimens. After staining with hematoxylin and eosin, the pathological sections were systematically examined by light microscopy to evaluate and document epidermal and dermal alterations. Periodic acid-Schiff (PAS) staining was performed when fungal infection was suspected. Acid-fast staining was performed when mycobacterial infection was included in the differential diagnosis.

### 2.5. Statistical Analysis

Statistical analyses were conducted using SPSS 27.0 and R 4.6.1 software. Continuous variables with non-normal distributions were expressed as medians and interquartile ranges, and categorical variables were summarized as frequencies and percentages. Comparisons of continuous variables among multiple independent groups were performed using the Kruskal–Wallis H test. Categorical variables were compared using Pearson’s chi-square test or Fisher’s exact test, as appropriate. For contingency tables larger than 2 × 2 with sparse expected counts, the Fisher–Freeman–Halton exact test was used.

Clinical outcomes were dichotomized as complete response and non-complete response, with partial response, stable disease, relapse, and death classified as non-complete response. One patient who died of myelodysplastic syndrome unrelated to the fungal infection was excluded from the prognosis analyses. To identify factors associated with non-complete response, univariable Firth logistic regression was performed to examine age (per 10-year increase), sex, disease duration (>12 versus ≤12 months), underlying conditions affecting host defense, lesion number (multiple versus solitary), and etiological category (dimorphic fungi as the reference group). Variables with *p* < 0.1 in univariable analysis were entered into the multivariable Firth logistic regression model. Odds ratios and 95% confidence intervals were reported. The apparent discrimination of the multivariable model was assessed using a receiver operating characteristic curve. In an analysis restricted to the 47 patients with sporotrichosis, age was compared using the Mann–Whitney U test, and complete-response rates were compared by clinical subtype, lesion site, itraconazole-containing treatment, potassium iodide-containing treatment, and host-defense status using Fisher’s exact or Fisher–Freeman–Halton exact tests as appropriate. *p* < 0.05 was considered statistically significant.

## 3. Results

### 3.1. General Data

A total of 86 patients were enrolled, including 35 males (40.7%) and 51 females (59.3%), with a male-to-female ratio of 0.69:1. The median age was 52.0 years (IQR, 39.3–64.0), ranging from 2 to 78 years. The disease duration ranged from 7 days to 20 years, with a median of 9.0 months (IQR, 4.0–24.0). Thirty-three patients (38.4%) had agricultural or other outdoor-related occupations, 19 (22.1%) reported a definite trauma history, and 22 (25.6%) had underlying conditions affecting host defense. Demographic and epidemiological characteristics are presented in Table 1.

### 3.2. Etiological Spectrum and Demographic Characteristics

Direct microscopy was performed in 31 patients and was positive in 8 patients (25.8%). Fungal elements observed by direct microscopy included hyphae, spores, pseudohyphae, or yeast-like spores.

**Figure 2:**
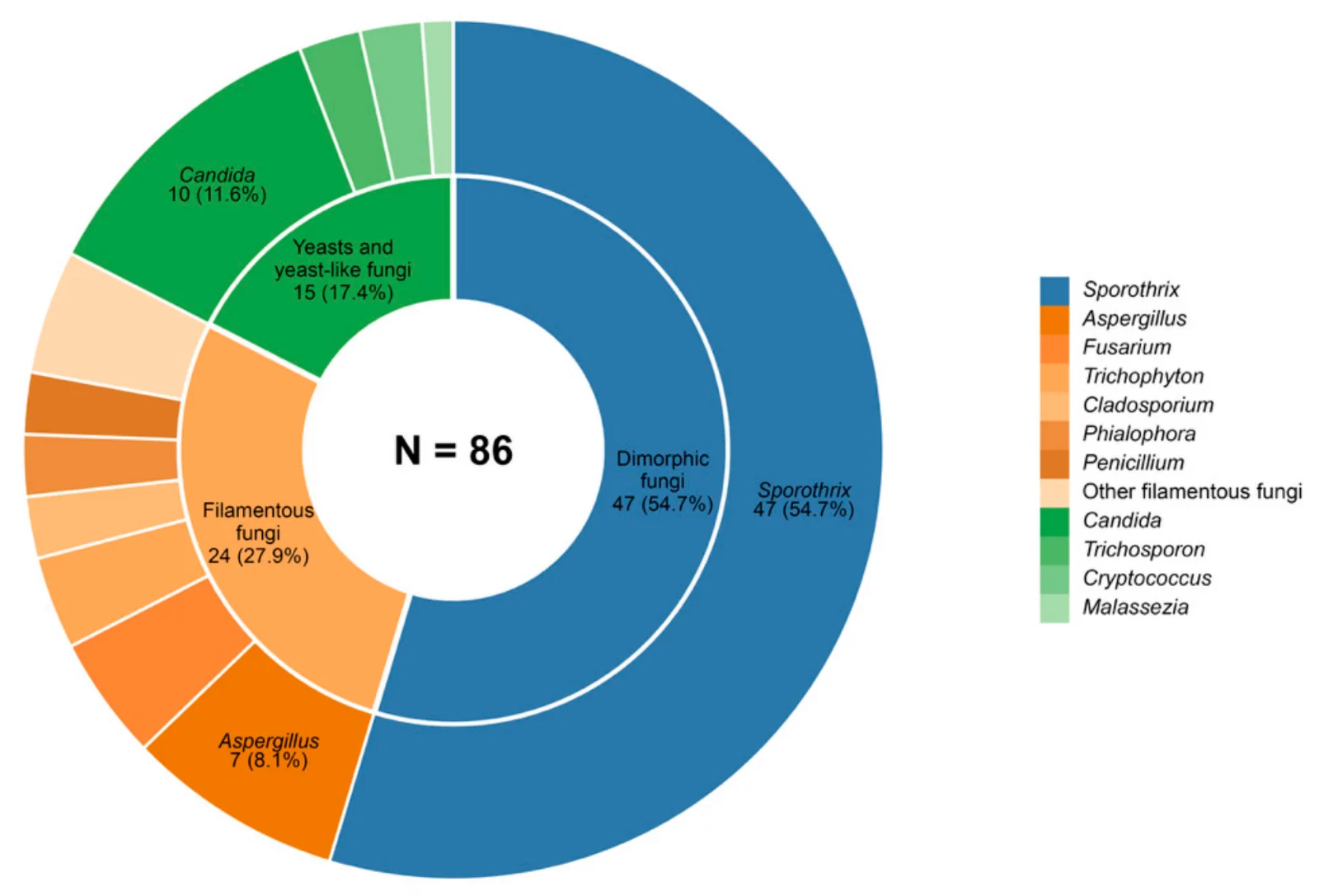
Etiological spectrum of subcutaneous mycoses in the study cohort. The inner ring shows the three major etiological categories, and the outer ring shows the genus-level distribution. Detailed pathogen and species distributions are provided in Table 2.

Fungal culture was positive in all 86 cases (100%). Dimorphic fungal infections accounted for 47 cases (54.7%), all caused by *Sporothrix* spp. Filamentous fungi accounted for 24 cases (27.9%), and yeasts and yeast-like fungi accounted for 15 cases (17.4%). Species and genus level distributions are shown in Figure 2 and Table 2.

Median age and the prevalence of underlying conditions affecting host defense differed significantly across the three etiological groups (*p* = 0.047 and *p* < 0.001, respectively), with the highest proportion of underlying conditions observed in the yeasts and yeast-like fungal group. Lower-limb involvement also differed among groups (*p* = 0.022). Disease duration, sex, and trauma history were not significantly different.

### 3.3. mNGS Findings

In the initially screened patients, mNGS was performed in 55 patients. Microbial sequences were detected in 42 patients (76.4%), whereas 13 patients (23.6%) had negative results. Bacterial sequences were detected in 37 patients (67.3%). In one patient, the cultured isolate was identified as *Trichosporon asahii* by ITS sequencing, whereas mNGS detected only low-read sequences of *Alternaria alternata*. Given the low read abundance and the lack of concordance with the culture-based molecular identification and clinical findings, the mNGS result was considered possible contamination. Four patients (7.3%) had clinically meaningful fungal findings. In two patients, the mNGS result was concordant with the culture result. In the other two patients, mNGS provided further species-level identification, identifying *Phialophora americana* from a culture reported at the genus level and *S. globosa* from a culture reported as *Sporothrix* spp.

### 3.4. Clinical Manifestations

**Figure 3:**
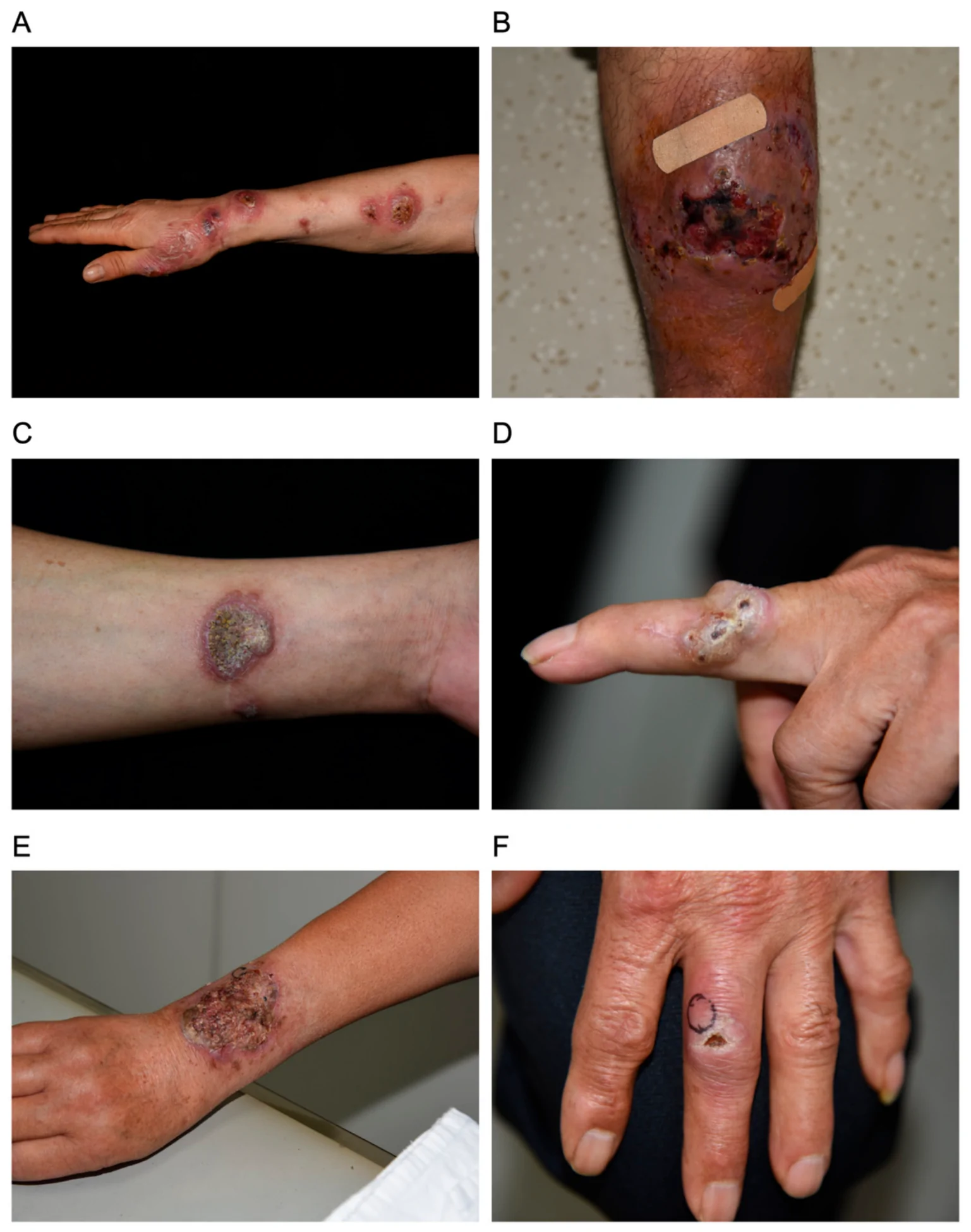
Representative clinical manifestations of subcutaneous mycoses caused by different fungal pathogens. Panel (**A**), lymphocutaneous sporotrichosis presenting as multiple erythematous nodules and ulcerated lesions distributed linearly along the upper limb. Panel (**B**), cutaneous fusariosis presenting as an ulcerated necrotic plaque on the lower leg. Panel (**C**), cutaneous infection caused by *Phialophora* spp., presenting as a solitary, well-demarcated verrucous and hyperkeratotic plaque. Panel (**D**), cutaneous candidiasis presenting as an ulcerated and crusted lesion on the finger. Panel (**E**), cutaneous aspergillosis presenting as an erythematous, verrucous, and crusted plaque on the wrist. Panel (**F**), cutaneous fusariosis presenting as a localized ulcerated lesion with central necrosis on the dorsal aspect of the finger (circle, biopsy site mark).

Clinical manifestations were heterogeneous, with the head and neck and upper limbs being the most frequently involved sites. Lower-limb involvement differed significantly among etiological groups (*p* = 0.022), whereas the major lesion morphologies did not. Among patients with sporotrichosis, 40/47 (85.1%) had fixed cutaneous disease and 7/47 (14.9%) had lymphocutaneous disease. Two patients had intracranial involvement, including one case caused by *Cryptococcus neoformans* and one caused by *Rhizopus* spp. Clinical manifestations of the 86 patients are summarized in Table 3. Representative clinical manifestations of subcutaneous mycoses caused by different fungal pathogens are shown in Figure 3.

### 3.5. Histopathological Characteristics.

**Figure 4:**
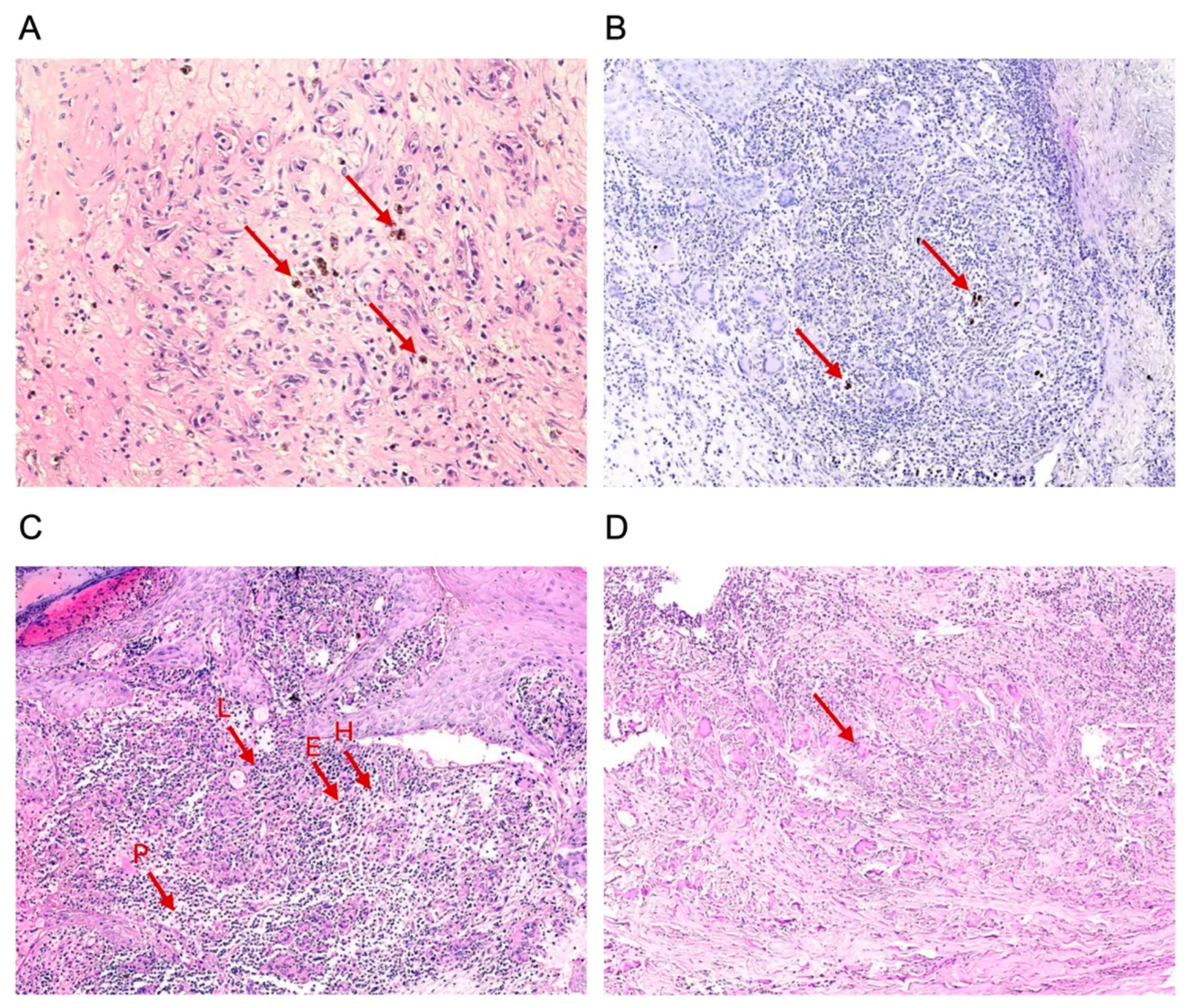
Representative histopathological findings in patients with subcutaneous mycoses. Panel (**A**), fungal spores (red arrows) identified within the dermal inflammatory infiltrate on hematoxylin and eosin staining. Panel (**B**), fungal elements (red arrows) highlighted by periodic acid-Schiff staining. Panel (**C**), dense mixed inflammatory cell infiltration in the dermis, comprising lymphocytes (L), plasma cells (P), histocytes (H), and eosinophils (E), on hematoxylin and eosin staining. Panel (**D**), multinucleated giant cells (red arrow) within the dermal inflammatory infiltrate on hematoxylin and eosin staining. Magnifications: (**A**) ×200, (**B**–**D**) ×40.

Skin histopathology was performed in 70 patients (81.4%). Mixed inflammatory infiltration was the predominant pattern (91.4%), whereas fungal structures were directly identified in only 8 patients (11.4%). Granulomatous inflammation and multinucleated giant cells differed significantly among etiological groups (*p* = 0.033 and *p* = 0.049, respectively), and were most frequent in the dimorphic fungal group. The histopathological findings are summarized in Table 4. Representative histopathological findings are shown in Figure 4.

### 3.6. Treatment and Outcomes

The most frequently used systemic antifungal agent was itraconazole, administered in 59 patients (68.6%). Potassium iodide was used in 36 patients (41.9%), fluconazole in 8 (9.3%), terbinafine in 4 (4.7%), and amphotericin B in 1 (1.2%). One patient received cryotherapy as adjunctive treatment. The median treatment duration was 3.0 months, ranging from 2 days to 24 months.

Prognosis analyses included 85 patients after excluding one patient who died of myelodysplastic syndrome unrelated to the fungal infection. Among the 85 patients, 72 (84.7%) achieved complete response, 7 (8.2%) achieved partial response, 2 (2.4%) had stable disease, 2 (2.4%) relapsed after complete response, and 2 (2.4%) died of fungal infection (Table 5). Complete response rates differed significantly among the three etiological groups. Complete response was achieved in 45 of 47 patients with dimorphic fungal infection (95.7%), 10 of 15 patients with yeasts and yeast-like fungal infection (66.7%), and 17 of 23 patients with filamentous fungal infection (73.9%, *p* = 0.004). Comparison of demographic, clinical, histopathological, and outcome characteristics among the three etiological groups is summarized in Table 6.

**Figure 5:**
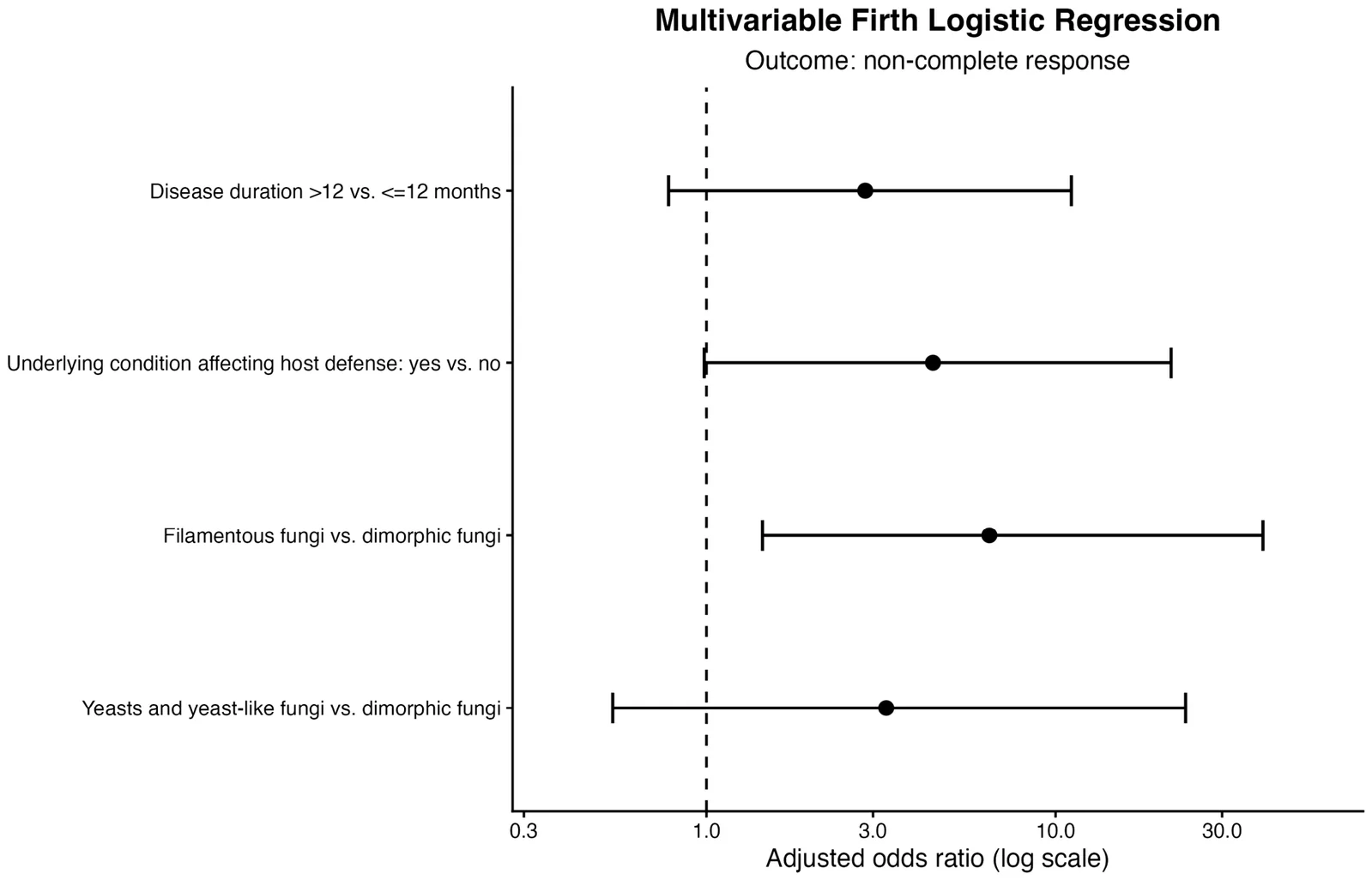
Forest plot of factors associated with non-complete response in patients with subcutaneous mycoses. Adjusted odds ratios (ORs) and 95% confidence intervals (CIs) were estimated using multivariable Firth logistic regression. Points represent adjusted ORs, horizontal lines indicate 95% CIs, and the vertical dashed line indicates the null value (OR = 1.0). The *x*-axis is displayed on a logarithmic scale. The reference categories were disease duration ≤ 12 months, absence of underlying conditions affecting host defense, and dimorphic fungal infection. Non-complete response was defined as partial response, stable disease, relapse after complete response, or death. OR, odds ratio; CI, confidence interval.

**Figure 6:**
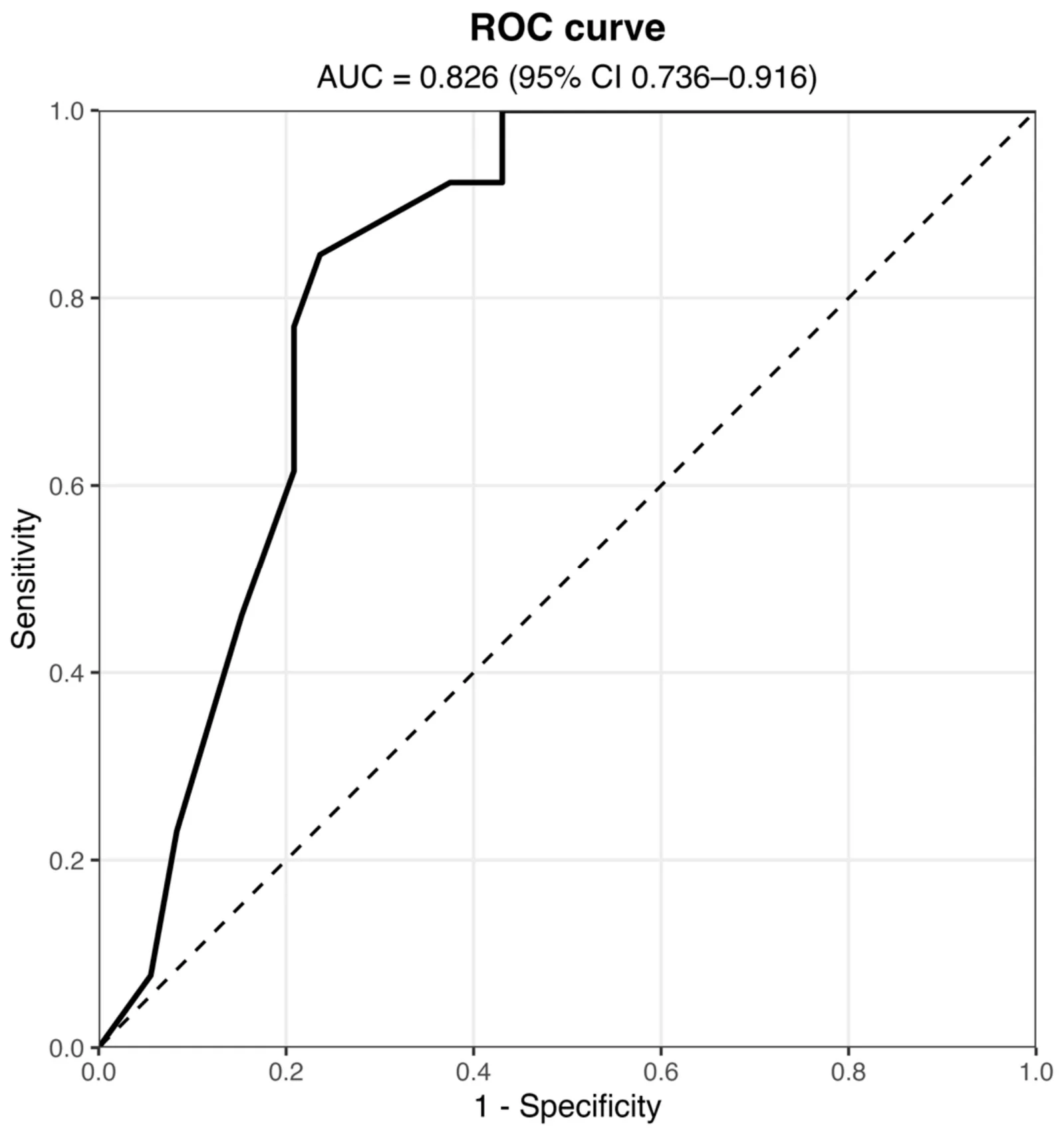
Receiver operating characteristic curve of the multivariable Firth logistic regression model for discriminating non-complete response in patients with subcutaneous mycoses. The area under the receiver operating characteristic curve was 0.826 (95% CI, 0.736–0.916). The solid line represents the ROC curve, and the dashed diagonal line represents no discrimination (AUC = 0.5). The *x*-axis indicates 1-specificity, and the *y*-axis indicates sensitivity. The curve reflects the apparent discrimination of the model in the study cohort of 85 patients, including 13 patients with non-complete response. Non-complete response was defined as partial response, stable disease, relapse after complete response, or death. ROC, receiver operating characteristic; AUC, area under the curve; CI, confidence interval.

Among the 21 patients with underlying conditions affecting host defense, 14 (66.7%) achieved complete response, compared with 58 of 64 patients (90.6%) without such conditions (*p* = 0.014). In univariable Firth logistic regression, underlying conditions affecting host defense, yeasts and yeast-like fungal infection, and filamentous fungal infection were associated with non-complete response. In the multivariable Firth model, filamentous fungal infection remained significantly associated with non-complete response compared with dimorphic fungal infection (OR, 6.46; 95% CI, 1.45–39.25; *p* = 0.014). Underlying conditions affecting host defense (OR, 4.46; 95% CI, 0.99–21.43; *p* = 0.052) and yeasts and yeast-like fungal infection (OR, 3.27; 95% CI, 0.54–23.57; *p* = 0.197) were not statistically significant after adjustment (Table 7, Figure 5). The apparent area under the receiver operating characteristic curve of the multivariable Firth logistic regression model was 0.826 (95% CI, 0.736–0.916) in the study cohort (Figure 6).

In the subgroup of 47 patients with sporotrichosis, all 41 patients (100%) without conditions affecting host defense achieved complete response, compared with 4 of 6 (66.7%) with such conditions (*p* = 0.014). No significant differences in response were observed by fixed versus lymphocutaneous subtype, age, lesion site, itraconazole-containing or potassium iodide-containing treatment (Supplementary Table S1).

## 4. Discussion

This 14-year retrospective study provides a comprehensive overview of subcutaneous mycoses in a tertiary referral center in Beijing. The cohort reveals a heterogeneous etiological spectrum in which sporotrichosis accounted for more than half of all cases. Most patients exhibited a chronic disease course, and approximately one-third of patients had experienced symptoms for longer than 12 months before diagnosis. This diagnostic delay reflects the indolent nature of these infections and highlights the diagnostic challenge posed by their nonspecific clinical manifestations. Chronic plaques, nodules, ulcers, verrucous lesions, and crusted lesions may mimic cutaneous tuberculosis, nontuberculous mycobacterial infection, chronic eczema, or cutaneous malignant tumors [18,19,20]. A study in eastern China reported a high misdiagnosis rate of 71.43% for sporotrichosis, with a mean time to correct diagnosis of 8.94 months [21]. Therefore, subcutaneous mycoses should be considered in the differential diagnosis of chronic or treatment-refractory lesions, particularly when lesions occur on exposed areas.

Subcutaneous mycoses are generally considered implantation fungal infections, usually resulting from the inoculation of environmental fungi into the dermis and subcutaneous tissues through trauma or minor skin injury [22,23]. However, only 22.1% of patients in our cohort reported a definite history of trauma, a proportion lower than that reported in previous studies of subcutaneous mycoses [4,5,24]. This may be because the subcutaneous lesions are indolent in onset with generally slow progression, and minor injuries or microtrauma can make it difficult for patients to recall. Therefore, the absence of a definite trauma history should not exclude the possibility of subcutaneous mycosis.

In this cohort, *Sporothrix* spp. was the predominant etiological agent, accounting for 54.7% of all cases. This differs from a recent retrospective study of primary subcutaneous mycoses in Shanghai, in which *Candida* spp. was the predominant pathogen group [5]. This geographical difference may reflect the unique epidemiological profile of China. Our institution in Beijing is geographically proximate to northeastern China, particularly Jilin Province, which is a hyperendemic region for sporotrichosis [10,25]. In contrast, Shanghai, as a coastal metropolis, may have different environmental exposures and a higher proportion of immunocompromised populations, leading to a higher prevalence of opportunistic yeast infections. Furthermore, climatic and seasonal factors also contribute to the regional epidemiology of sporotrichosis. In northeast China, the higher incidence during winter and spring has been linked to increased exposure to decaying vegetation, particularly contaminated cornstalks that are stored or used as biomass fuel during colder months [26,27]. The predominance of *S. schenckii* over *S. globosa* should be interpreted cautiously because identification methods changed during the 14-year study period. Historically, isolates were often reported as *S. schenckii* sensu lato, whereas molecular methods are required to reliably distinguish members of the *S. schenckii* complex [28,29]. Thus, the observed species distribution may partly reflect historical reporting practices.

Most patients with sporotrichosis in our cohort presented with fixed cutaneous disease, whereas lymphocutaneous sporotrichosis accounted for a smaller proportion, and no disseminated cutaneous sporotrichosis was observed. This clinical pattern is consistent with the epidemiology observed in endemic areas such as Jilin Province [10]. A 20-year retrospective study from eastern China also found the fixed form (69.39%) to be more common than the lymphocutaneous form [21]. In contrast, lymphocutaneous sporotrichosis is more frequently reported in many different countries [30]. In Brazil, sporotrichosis is frequently caused by *S. brasiliensis* and is often associated with zoonotic transmission through infected cats [31,32]. This difference may be because the routes of transmission, the size and depth of inoculation, the genotype of the fungus, and the immune status of the host can influence the clinical presentation [33,34]. It has been hypothesized that the fixed cutaneous form reflects a higher level of host immunity against *Sporothrix* spp., possibly acquired through frequent exposure to fungal antigens in endemic regions [35,36].

In addition to *Sporothrix* spp., our study identified a wide range of yeasts, yeast-like fungi, molds, and dematiaceous fungi, indicating a heterogeneous etiological spectrum. Although *Sporothrix* spp. remains dominant, opportunistic fungi and uncommon molds can also cause subcutaneous infection, especially in immunocompromised hosts. Host factors, particularly immune status, play an important role in the occurrence and outcome of subcutaneous fungal infections. Previous studies have reported that diabetes mellitus, HIV infection, hematological malignancy, solid organ transplantation, long-term use of corticosteroid or immunosuppressive therapy, and the use of broad-spectrum antimicrobial therapy may increase susceptibility to infection and are associated with a high risk of dissemination, recurrence, or treatment difficulty [5,37,38]. In our cohort, underlying conditions affecting host defense were present in 25.6% of patients, significantly more frequent in the yeasts and yeast-like fungal group (73.3%). The study from Shanghai also reported an increasing number of primary subcutaneous mycoses and suggested a possible association with immune deficiency or immunosuppressive therapy [5]. These findings suggest that host immune status may influence the etiological spectrum and highlight the importance of considering *Candida* spp. and other opportunistic fungi in immunocompromised patients.

Fungal culture remained the cornerstone of etiological diagnosis in this study, providing crucial evidence for pathogen identification and guiding treatment. In contrast, direct microscopy was positive in only 25.8% of examined patients. This relatively low positive rate indicates that negative direct microscopy cannot rule out subcutaneous mycoses, particularly in infections with low fungal burden such as sporotrichosis [39,40]. Culture is still essential because it allows organisms to be recovered and identified and enables antifungal susceptibility testing. However, culture may be affected by specimen quality, fungal load, prior antifungal therapy, slow growth, or contamination [11,41]. In addition to conventional culture, molecular techniques including polymerase chain reaction (PCR), internal transcribed spacer (ITS)-based sequencing, and mNGS, have important complementary value in the early diagnosis and etiological identification of subcutaneous mycoses. These techniques can reduce diagnostic turnaround time, improve detection in cases with low fungal burden, culture-negative cases, or cases previously treated with antifungal agents, and facilitate species-level identification. mNGS has shown potential value in difficult, mixed, or culture-negative skin and soft-tissue infections because it can simultaneously detect bacteria, fungi, viruses, parasites, and other pathogens. However, molecular testing still has several limitations, including high cost, background interference from host nucleic acids, risk of contamination, and difficulty in distinguishing colonization or contamination from true infection [42,43,44]. Therefore, an integrated assessment combining mycological, histopathological, and molecular methods is important for improving the diagnosis of subcutaneous mycoses.

In our cohort, the predominant pattern was mixed inflammatory cell infiltration, frequently accompanied by epidermal hyperplasia, ulceration, granulomatous inflammation, and multinucleated giant cells. This pattern is consistent with chronic infectious inflammation in subcutaneous fungal infections, in which histopathology helps evaluate tissue reaction, fungal localization, and possible tissue invasion. Notably, granulomatous inflammation and multinucleated giant cells were more frequent in the dimorphic fungal group. This finding is consistent with previous observations that sporotrichosis commonly presents with granulomatous or suppurative-granulomatous inflammation, while fungal elements may be difficult to detect in tissue sections [16]. However, the pathological pattern alone was not specific for a specific pathogen in many cases. In addition, fungal elements were directly observed in only a small proportion of cases, and PAS staining was positive in only a minority of tested cases. Therefore, although histopathology is useful for supporting the diagnosis and evaluating tissue reaction, it is often not sufficient for pathogen identification. Based on these complementary strengths and limitations, we propose an integrated three-tiered diagnostic strategy for subcutaneous mycoses. Histopathological examination may serve as an early direction tool by providing preliminary diagnostic clues, revealing pathological and inflammatory patterns, and assessing tissue invasion, although it is limited by low sensitivity and sampling bias. Fungal culture remains the confirmatory reference method, enabling etiological identification, antifungal susceptibility testing, and strain preservation, but requires a prolonged turnaround time. mNGS may serve as a problem-solving tool for challenging cases because of its broad-spectrum detection capability, potential for species-level identification, and ability to detect mixed infections, although its high cost and susceptibility to contamination require expert interpretation. This integrated strategy may improve diagnostic accuracy and reduce delays in therapeutic decision-making.

In the present cohort, systemic antifungal therapy was the mainstay of treatment, with itraconazole being the most frequently used agent, followed by potassium iodide, which was mainly administered for sporotrichosis. The overall treatment response in our cohort was favorable, with 84.7% of patients achieving complete response. This may be partly explained by the predominance of *Sporothrix* spp. infection and the high proportion of fixed cutaneous sporotrichosis, which is usually localized and responds well to oral antifungal therapy [45]. However, the complete response rate differed significantly among the three etiological groups, suggesting that empirical therapy may be better pathogen-stratified. In immunocompetent patients in this region, *Sporothrix* spp. is the predominant pathogen, and itraconazole or potassium iodide may be used as first-line empirical therapy. However, in patients with underlying comorbidities affecting host defense, filamentous fungi and yeasts may be suspected, and broader-spectrum agents may be considered upfront, as these groups have significantly lower complete response rates (73.9% and 66.7%, respectively) than *Sporothrix* spp. (95.7%, *p* = 0.004), and filamentous fungi infection is an independent risk factor for non-complete response (OR = 6.46, *p* = 0.014). Furthermore, patients with immunosuppression may have a different spectrum of causative pathogens and worse clinical outcomes. Underlying conditions affecting host defense were present in 73.3% of patients with yeasts and yeast-like fungal infections, in contrast with 12.8% of those with dimorphic fungal infections and 20.8% of those with filamentous fungal infections (*p* < 0.001). The overall complete-response rate in patients with underlying conditions affecting host defense was also significantly lower than that in patients without such conditions (66.7% vs. 90.6%, *p* = 0.014). Importantly, host factors also appeared to influence outcome even within the same etiological group. In the analysis restricted to patients with sporotrichosis, those with underlying conditions affecting host defense had a lower complete-response rate than those without such conditions. These findings suggest that patients with underlying immunosuppression constitute a high-risk subgroup that requires intensified monitoring and more individualized treatment, even when infected with an otherwise highly treatment-responsive pathogen such as *Sporothrix* spp.

The present study has several limitations. First, this was a retrospective single-center study, and the completeness of data depended on medical records and follow-up information. Second, not all patients underwent the same diagnostic procedures, including direct microscopy, mNGS, histopathology, PAS staining, and antifungal susceptibility testing. Third, several pathogens were identified only to the genus level, which may limit more detailed analysis of species distribution. Fourth, the follow-up duration varied among patients, and long-term recurrence may have been underestimated.

## 5. Conclusions

This study demonstrates the considerable clinical and etiological diversity of subcutaneous mycoses in a tertiary referral center in China, with *Sporothrix* spp. being the predominant pathogen. Most patients achieved favorable outcomes; however, filamentous fungal infections were significantly associated with an increased likelihood of non-complete response. An integrated diagnostic strategy of histopathology, fungal culture and mNGS is recommended for subcutaneous mycoses. Empirical therapy may be better pathogen-stratified. Patients with underlying immunosuppression constitute a high-risk subgroup that requires intensified monitoring and more aggressive treatment.

## Figures and Tables

**Table 1:** Demographic and epidemiological characteristics of the 86 patients.

Characteristic	Category	Value
Sex	Male	35 (40.7%)
	Female	51 (59.3%)
Age, years	Median (IQR; range)	52.0 (39.3–64.0; 2–78)
	<18	8 (9.3%)
	18–30	7 (8.1%)
	31–40	10 (11.6%)
	41–50	14 (16.3%)
	51–60	19 (22.1%)
	61–70	20 (23.3%)
	>70	8 (9.3%)
Disease duration, months	Median (IQR; range)	9.0 (4.0–24.0; 0.23–240)
Occupation	Agricultural/Forestry/Gardening	29 (33.7%)
	Animal-related	2 (2.3%)
	Other outdoor work	2 (2.3%)
	Indoor work	53 (61.6%)
Trauma history	Puncture injuries by wood or plant materials	3 (3.5%)
	Acupuncture	2 (2.3%)
	Nail puncture	1 (1.2%)
	Dog scratch	1 (1.2%)
	Insect bite	1 (1.2%)
	Burns	1 (1.2%)
	Acne squeezing	1 (1.2%)
	Cosmetic procedures	1 (1.2%)
	Tattooing	1 (1.2%)
	Other trauma events	7 (8.1%)
Underlying conditions affecting host defense	Total	22 (25.6%)
	Diabetes mellitus	11 (12.8%)
	Systemic corticosteroid or other immunosuppressant therapy	10 (11.6%)
	Hematological malignancy	3 (3.5%)
	Immunodeficiency disease	1 (1.2%)

**Table 2:** Pathogen distribution.

Pathogen Category	Genus	Species	Number of Cases
Dimorphic fungi (*n* = 47)	genus *Sporothrix* (*n* = 47)	*Sporothrix schenckii*	43
		*Sporothrix globosa*	2
		*Sporothrix* spp.	2
Filamentous fungi (*n* = 24)	genus *Aspergillus* (*n* = 7)	*Aspergillus fumigatus*	1
		*Aspergillus terreus*	1
		*Aspergillus flavus*	1
		*Aspergillus nidulans*	1
		*Aspergillus glaucus*	1
		*Aspergillus* spp.	2
	genus *Fusarium* (*n* = 4)	*Fusarium solani*	1
		*Fusarium* spp.	3
	genus *Trichophyton* (*n* = 3)	*Trichophyton rubrum*	3
	genus *Cladosporium* (*n* = 2)	*Cladosporium* spp.	2
	genus *Phialophora* (*n* = 2)	*Phialophora verrucosa*	1
		*Phialophora americana*	1
	genus *Penicillium* (*n* = 2)	*Penicillium* spp.	2
	genus *Microsporum* (*n* = 1)	*Microsporum canis*	1
	genus *Alternaria* (*n* = 1)	*Alternaria* spp.	1
	genus *Rhizopus* (*n* = 1)	*Rhizopus* spp.	1
	genus Unidentified filamentous fungus (*n* = 1)	Unidentified filamentous fungus	1
Yeasts and yeast-like fungi (*n* = 15)	genus *Candida* (*n* = 10)	*Candida albicans*	4
		*Candida parapsilosis*	4
		*Candida guilliermondii*	1
		*Candida* spp.	1
	genus *Trichosporon* (*n* = 2)	*Trichosporon asahii*	1
		*Trichosporon* spp.	1
	genus *Cryptococcus* (*n* = 2)	*Cryptococcus neoformans*	2
	genus *Malassezia* (*n* = 1)	*Malassezia* spp.	1

**Table 3:** Clinical characteristics of the 86 patients.

Characteristic	Category	*n* (%)
Lesion number	Solitary	61 (70.9%)
	Multiple	25 (29.1%)
Anatomical site	Head and neck	38 (44.2%)
	Upper limbs	33 (38.4%)
	Lower limbs	16 (18.6%)
	Trunk	4 (4.7%)
Lesion morphology	Erythematous patch/plaque	56 (65.1%)
	Nodule/mass	35 (40.7%)
	Ulceration/erosion	30 (34.9%)
	Papule	19 (22.1%)
Sporotrichosis subtype	Fixed cutaneous	40/47 (85.1%)
	Lymphocutaneous	7/47 (14.9%)
Deep organ involvement	Present	2 (2.3%)

**Table 4:** Histopathological findings.

Histopathological Characteristics	*n* (%)
Epidermal changes	
Acanthosis, epidermal hyperplasia, or elongation of the rete ridges	50 (71.4%)
Superficial crusting, epidermal disruption, or ulceration	31 (44.3%)
Pseudoepitheliomatous hyperplasia	19 (27.1%)
Dermal changes	
Mixed inflammatory cell infiltration	64 (91.4%)
Lymphocytic infiltration	64 (91.4%)
Plasma cell infiltration	57 (81.4%)
Histiocytic infiltration	48 (68.6%)
Neutrophilic infiltration	42 (60.0%)
Eosinophilic infiltration	10 (14.3%)
Granulomatous inflammation	33 (47.1%)
Multinucleated giant cells	39 (55.7%)
Pathogen-related findings	
Fungal structures observed in tissue	8 (11.4%)
Hyphae	4 (5.7%)
Spores	4 (5.7%)
Thick-walled spores	1 (1.4%)
Asteroid body	1 (1.4%)
PAS staining performed	14 (20.0%)
Positive PAS staining	4/14 (28.6%)

**Table 5:** Treatment and outcomes.

Category	Variable	*n* (%)
Systemic treatment	Itraconazole	59 (68.6%)
	Potassium iodide	36 (41.9%)
	Fluconazole	8 (9.3%)
	Terbinafine	4 (4.7%)
	Amphotericin B	1 (1.2%)
Adjunctive treatment	Cryotherapy	1 (1.2%)
Treatment duration	Median treatment duration, months	3.0
	Range	2 days-24 months
Clinical outcome (*n* = 85)	Complete response	72 (84.7%)
	Partial response	7 (8.2%)
	Stable disease	2 (2.4%)
	Relapse after complete response	2 (2.4%)
	Death	2 (2.4%)

**Table 6:** Comparison of demographic, clinical, histopathological, and outcome characteristics among fungal etiological groups.

Characteristic	Dimorphic Fungi (*n* = 47)	Filamentous Fungi (*n* = 24)	Yeasts and Yeast-Like Fungi (*n* = 15)	*p* Value
Demographics				
Age, median (IQR), years	57.0 (48.5–64.5)	47.5 (28.8–57.8)	49.0 (30.5–62.0)	0.047
Disease duration, median (IQR), months	6.0 (4.0–14.0)	12.0 (5.5–36.0)	18.0 (9.0–30.0)	0.113
Male, *n* (%)	14 (29.8)	14 (58.3)	7 (46.7)	0.060
Trauma history, *n* (%)	13 (27.7)	4 (16.7)	2 (13.3)	0.463
Underlying conditions affecting host defense, *n* (%)	6 (12.8)	5 (20.8)	11 (73.3)	<0.001
Cutaneous involvement, *n* (%)				
Head and neck	24 (51.1)	10 (41.7)	4 (26.7)	0.243
Upper limb	17 (36.2)	8 (33.3)	8 (53.3)	0.412
Lower limb	4 (8.5)	7 (29.2)	5 (33.3)	0.022
Trunk	2 (4.3)	1 (4.2)	1 (6.7)	1.000
Lesion morphology, *n* (%)				
Plaque	28 (59.6)	17 (70.8)	11 (73.3)	0.490
Nodule	22 (46.8)	8 (33.3)	5 (33.3)	0.449
Ulcer	17 (36.2)	8 (33.3)	5 (33.3)	0.963
Papule	12 (25.5)	5 (20.8)	2 (13.3)	0.602
Histopathology (*n* = 70 biopsies)	(*n* = 40)	(*n* = 17)	(*n* = 13)	
Granulomatous inflammation, *n* (%)	24 (60.0)	4 (23.5)	5 (38.5)	0.033
Multinucleated giant cells, *n* (%)	27 (67.5)	8 (47.1)	4 (30.8)	0.049
Outcome, *n* (%)				
Complete response	45 (95.7)	17 (73.9)	10 (66.7)	0.004

One patient in the filamentous fungal group who died of an unrelated disease was excluded from the outcome analysis.

**Table 7:** Factors associated with non-complete response in subcutaneous mycoses.

Factors	Comparison	Univariable Analysis			Multivariable Analysis		
		OR	95% CI	*p*	OR	95% CI	*p*
Age	per 10-year increase	0.94	0.71–1.28	0.676			
Sex	male vs. female	1.91	0.60–6.24	0.271			
Disease duration	>12 vs. ≤12 months	2.76	0.86–9.15	0.088	2.85	0.78–11.10	0.113
Underlying conditions affecting host defense	yes vs. no	4.66	1.41–15.96	0.012	4.46	0.99–21.43	0.052
Lesion number	multiple vs. solitary	1.78	0.51–5.78	0.353			
Etiological category	Dimorphic fungi (reference)	1.00 (reference)			1.00 (reference)		
	Filamentous fungi	6.76	1.55–39.70	0.010	6.46	1.45–39.25	0.014
	Yeasts and yeast-like fungi	9.53	1.99–59.46	0.005	3.27	0.54–23.57	0.197

OR, odds ratio; CI, confidence interval. Non-complete response included partial response, stable disease, relapse after complete response, and death.

## Data Availability

The data presented in this study are available on request from the corresponding author due to patient privacy and institutional ethical restrictions.

## Supplementary Materials

The following supporting information can be downloaded at: https://www.mdpi.com/article/10.3390/jof12090692/s1, Figure S1: Representative microscopic morphologies of cultured fungal isolates identified in patients with subcutaneous mycoses; Table S1: Subgroup analysis of complete response among 47 patients with sporotrichosis.
