# Supplementary material for: Epidemiology, Clinicopathological Features, Etiological Spectrum and Outcomes of Subcutaneous Mycoses: A 14-Year Retrospective Study
Source: J Fungi (Basel). 2026 Sep 16;12(9):692. doi: 10.3390/jof12090692 (PMC13608034; doi:10.3390/jof12090692)
Supplement: Supplementary file 1 [file jof-12-00692-s001.zip › jof-4513917-supplementary.pdf]

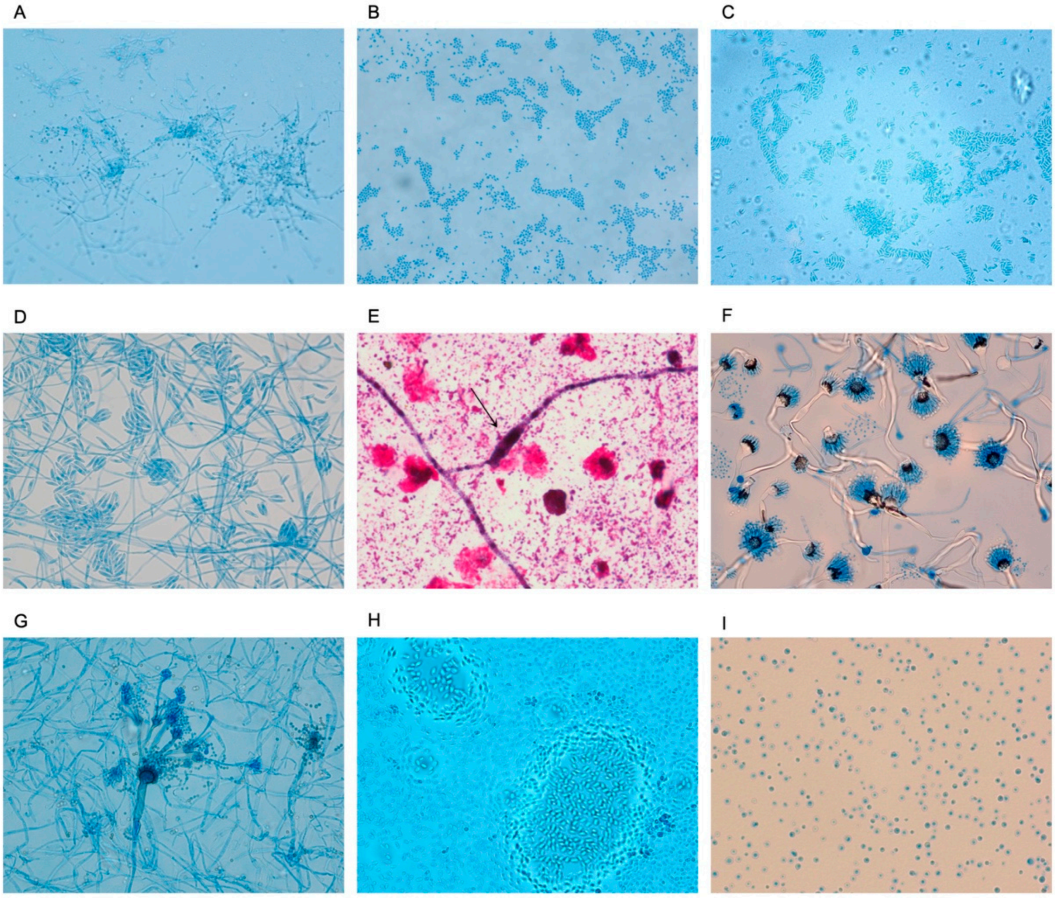

Figure S1. Representative microscopic morphologies of cultured fungal isolates identified in patients with subcutaneous mycoses. Panel A, mycelial phase of *Sporothrix*. Panel B, yeast phase of *Sporothrix*. Panel C, *Phialophora*. Panel D and E, *Fusarium*. Panel F, *Aspergillus terreus*. Panel G, *Aspergillus fumigatus*. Panel H, *Exophiala*. Panel I, *Cryptococcus*.

Table S1. Subgroup analysis of complete response among 47 patients with sporotrichosis

Factor	Category	Complete response	P value
Age, years	Complete vs. non-complete response	55.0 (IQR, 48.0-64.0) vs. 71.0 (68-74)	0.073
Clinical subtype	Fixed cutaneous	38/40 (95.0%)	1.000
	Lymphocutaneous	7/7 (100%)	
Anatomic site	Head and neck	23/24 (95.8%)	0.241
	Upper limbs	17/17 (100%)	
	Lower limbs	3/4 (75.0%)	
	Trunk	2/2 (100%)	
Itraconazole-containing treatment	Yes	30/31 (96.8%)	1.000
	No	15/16 (93.8%)	
Potassium iodide-containing treatment	Yes	32/33 (97.0%)	0.512
	No	13/14 (92.9%)	
Underlying conditions affecting host defense	Yes	4/6 (66.7%)	0.014
	No	41/41 (100%)	
